# Functional fermented dairy products: a review of mechanisms, health potential, and technological challenges

**DOI:** 10.3389/fnut.2026.1779688

**Published:** 2026-05-15

**Authors:** Beyza Nur Arslan, Naciye Yaktubay Döndaş, Senanur Koçhan, Sena Davran Bulut, Mehmet Ali Tamer, H. Ufuk Çelebioğlu, Mahmut Bodur, Duygu Ağagündüz, Giulia Tabanelli, Federica Barbieri, Fausto Gardini, H. Ali Döndaş, Tuba Esatbeyoglu, Fatih Ozogul

**Affiliations:** 1 Department of Medical Pharmacology, Faculty of Medicine, Cukurova University, Adana, Türkiye; 2 Department of Basic Pharmaceutical Sciences, Faculty of Pharmacy, Çukurova University, Adana, Türkiye; 3 Department of Biotechnology, Institute of Natural and Applied Sciences, Çukurova University, Adana, Türkiye; 4 Department of Translational Medicine, Institute of Health Sciences, Çukurova University, Adana, Türkiye; 5 Department of Biotechnology, Faculty of Science, Bartin University, Bartin, Türkiye; 6 Department of Nutrition and Dietetics, Faculty of Health Sciences, Ankara University, Ankara, Türkiye; 7 Department of Nutrition and Dietetics, Faculty of Health Sciences, Gazi University, Ankara, Türkiye; 8 Department of Agricultural and Food Sciences, University of Bologna, Bologna, Italy; 9 Department of Agricultural and Food Sciences, University of Bologna, Cesena, Italy; 10 Department of Advanced Materials and Nanotechnology, Institute of Natural and Applied Sciences, Cukurova University, Adana, Türkiye; 11 Department of Molecular Food Chemistry and Food Development, Institute of Food and One Health, Gottfried Wilhelm Leibniz University Hannover, Hannover, Germany; 12 Department of Seafood Processing Technology, Faculty of Fisheries, Cukurova University, Adana, Türkiye; 13 Biotechnology Research and Application Center, Cukurova University, Adana, Türkiye

**Keywords:** bioactive peptides, dairy fermentations, functional foods, gut microbiota, lactic acid bacteria, probiotics

## Abstract

Fermented dairy products such as yoghurt, kefir and cheese are increasingly recognised as functional foods due to the metabolic activity of lactic acid bacteria and the associated microbial communities, including probiotics. During dairy fermentation, these microorganisms generate bioactive compounds, such as bioactive peptides, exopolysaccharides, organic acids and other metabolites, which may contribute to host health. There is emerging evidence that fermented dairy products can influence gastrointestinal function, immune regulation, metabolic health and cardiovascular risk, via mechanisms involving modulation of the gut microbiota, stabilisation of the epithelial barrier and inflammatory signalling pathways. In addition, fermentation may improve lactose digestion, enhance nutrient bioavailability, and generate peptides with anti-hypertensive or antioxidant properties. However, translating these results into consistent health benefits is challenging due to the significant variability in microbial strains, product composition, processing conditions and dosage. Safety considerations such as biogenic amines, sodium content, allergenicity and antimicrobial resistance also require careful monitoring. Future progress in this field will depend on improved product characterisation, strain-level identification and well-designed human intervention studies that integrate multi-omics approaches. In conclusion, fermented dairy products show great potential as a source of bioactive compounds, but more robust clinical evidence and standardised methodologies are required to firmly establish their role in promoting human health.

## Graphical abstract

**Figure:**
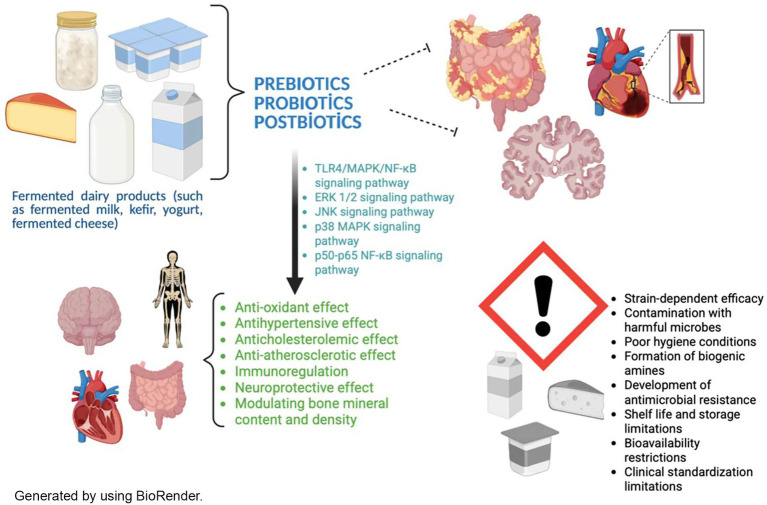
(Generated by using BioRender).

## 1 Introduction

Milk is the first food of mammals and a nutrient-dense matrix providing macronutrients, vitamins, minerals, and immunologically relevant factors. From a physicochemical perspective, it is a complex multiphase system: lactose, whey proteins, minerals, and small solutes reside in an aqueous phase; hydrophobic caseins form colloidal micelles stabilised by *κ*-casein; and triacylglycerol-rich fat globules are emulsified by a membrane of proteins and phospholipids (1). Despite innate antimicrobial defences (lysozyme, lactoferrin, lactoperoxidase), raw milk readily supports microbial growth owing to near-neutral pH (~6.6), high water activity (~0.99), and warm milking temperature (~37 °C) (1).

Before reliable cold chains became available in the late nineteenth century, milk’s short shelf-life necessitated preservation. Alongside salting and drying, fermentation emerged millennia ago as a robust strategy to extend shelf life, improve safety, and enhance quality, with early evidence from civilisations between the Tigris and Euphrates (2–4). Empirical practices such as back-slopping and the use of natural microbial consortia evolved into starter-culture technologies; storage in animal-derived pouches likely provided microbial reservoirs and informed rennet use (2, 3). The use of different milk sources, microbial communities, and processing technologies has resulted in a broad diversity of culturally distinctive dairy products across geographical regions and historical periods, including yogurt, kefir, and a wide variety of cheeses (2, 4).

Fermented foods are defined as products that, at some stage of production, require guided activity of specific microorganisms at high concentrations and that, relative to the raw material, exhibit improved attributes such as extended shelf life, enhanced nutritional properties, greater palatability, and reduced hygiene and health risks (Marco et al., 2021). In dairy systems, lactic acid bacteria (LAB) and adjunct microbes acidify the matrix, promote coagulation, shape flavour and texture, and generate bioactive metabolites with potential nutritional and physiological effects (2, 4). The fermented dairy sector (cheese, yogurt, butter, sour cream, kefir, buttermilk, fermented milk drinks) reached USD 292.5 billion in 2023, with a projected 2024–2032 CAGR of 5.1%, driven by demand for probiotic and functional products (5). At the same time, the microbial growth suitability of milk underscores the need for rigorous hygiene from milking onward (2, 6). This review therefore examines fermented dairy products and discusses how fermentation processes, microbial ecology, and strain-level characteristics influence the formation of bioactive compounds with potential implications for human health, while also addressing technological challenges, product variability, and current limitations in the available evidence.

## 2 Types of dairy-based fermented products

### 2.1 Overview of dairy fermentation processes

As already noted, milk is a complex system in which, from a chemical and physical point of view, three phases coexist: an aqueous solution, a colloidal suspension and an emulsion. Microorganisms involved in dairy fermentation processes, which are primarily aimed at extending the shelf life of milk or its derivatives, play a fundamental role, together with physicochemical and technological factors, in modifying and destabilizing the native milk system. Three types of products can be obtained from milk processing: butter, fermented milks and cheese. Although butter can also be obtained from pre-fermented sour creams (7), in this review, it was decided to consider only those products in which fermentation plays a more central role.

Lactic acid bacteria (LAB) are the principal drivers of acidification in most dairy fermentations during the first steps of cheese making. They are gram-positive, non-spore forming bacteria that obtain the energy they need from fermentative metabolisms. The two primary metabolic pathways of LAB are homolactic fermentation (leading to the formation of only lactic acid as an end product, in the form of D-lactate, L-lactate or their racemic mixture, depending on the genus considered) and heterolactic fermentation (with the formation of CO_2_, lactic acid and ethanol or acetic acid). The most important species are, of course, those capable of exploiting the carbohydrate resource present in milk, namely lactose (2).

Whatever pathway is adopted by LAB, the result of their activity on the environment (milk) consists primarily of lowering pH. The increase in acidity, in addition to having a counteracting action toward undesirable microorganisms (comprising spoilers and pathogens), plays an important role toward caseins present in the form of colloidal suspension, destabilizing them. In fact, on the one hand, the decrease in pH reduces the hydrophilic capacity of k-casein until it is nullified, when the isoelectric point is reached (approximately pH 4.6). At this value in fact, no electric charge is present in k-casein molecules (and ultimately on casein micelles) and they lose the capacity to interact with water and their suspension status. In addition, on the other hand, pH decrease solubilizes the tricalcium phosphate that acts as a cementing agent between the different caseins present in the individual micelles.

In addition to these primary metabolic pathways, several other LAB metabolisms contribute to the characterisation of dairy products (8). First, certain enzymatic activities (primarily proteolytic enzymes and peptidases, but also esterases) can influence the textural, organoleptic and even nutritional characteristics of the finished product (9). Equally important is the ability of certain microorganisms to produce exopolysaccharides, including both homopolysaccharides (i.e., *α*-glucans, *β*-glucans, β-fructans, and β-galactans) and heteropolysaccharides (kefiran). These polymers significantly influence the water-holding capacity and viscosity of fermented dairy products and may also confer relevant nutritional and functional properties (10).

Moreover, the activation of secondary metabolic pathways beyond classical homolactic or heterolactic fermentation is of considerable relevance. These include mixed end-product pathways involving pyruvate formate-lyase and lactate decarboxylase, the conversion of pyruvate via the α-acetolactate pathway, and amino acid catabolism. Collectively, these metabolic routes contribute significantly to the development of the sensory characteristics of fermented products and may also influence their nutritional value (11–13). The ability of some LAB to produce antimicrobial substances, such as bacteriocins or peptides and other organic substances capable of inhibiting or otherwise limiting the growth of undesirable microorganisms, should also not be underestimated (Alvarez-Sieirio et al., 2016) (14).

Many LAB genera (and related species) are involved in dairy fermentations throughout the world. For their long history of safe use, they have been included in the list of Qualified Presumption of Safety (QPS) by European Food Safety Authority (EFSA) since its introduction (15). Among thermophilic homofermentative lactobacilli, the species more commonly involved are *Lactobacillus helveticus*, *Lactobacillus delbrueckii* (with the subspecies *lactis* and *bulgaricus*) *Lactobacillus acidophilus*, *Lactobacillus johnsonii*. Among facultatively heterofermentative LAB*, Lacticaseibacillus casei*, *Lacticaseibacillus paracasei*, *Lacticaseibacillus rhamnosus*, *Lactiplantibacillus plantarum*, *Lactiplantibacillus paraplantarum*, *Latilactibacillus curvatus*, *Lactiplantibacillus pentosus* are among the common species found in dairy products. Finally, among obligate heterofermentative species, the most involved in dairy fermentation are *Levilactobacillus brevis, Lentilactobacillus buchneri*, and *Limosilactobacillus fermentum*. Taking into consideration cocci, the species more used are the homofermentative *Streptococcus thermophilus*, *Lactococcus lactis* and the heterofermentative species *Leuconostoc mesenteroides* (16–19).

Although many strains of these species have been selected as starter cultures in the production of dairy products since the early 20th century, in many cases, the use of natural complex cultures capable of initiating fermentations is still used, both for cheese and fermented milk production (8).

In addition to lactic acid bacteria, other microbial species also contribute to the production of fermented dairy foods. For example, propionibacteria (*Propionibacterium freudenrichii*) play a role, as do acetic bacteria (usually *Acetobacter* spp.) in some fermented milks. In the production of some dairy products, fungi and in particular yeasts, such as *Kluyveromyces marxianus*, or molds, such as *Penicillium roqueforti, Penicillium camemberti*, and *Geotrichum candidum,* are also present (20).

### 2.2 Fermented milks

Fermented milks are obtained through the coagulation of milk by a decrease in pH induced by microbial activity, without the removal of whey. Hundreds of different types of fermented milks are known to be consumed worldwide (6). Fermented milks are often regarded as functional foods, as fermentation not only preserves many essential milk nutrients for a longer period than the raw material but also modifies certain milk components in ways that enhance their nutritional value. At the same time, they provide consumers with significant amounts of live, active cultures, offering health benefits beyond basic nutrition and serving as an effective vehicle for the delivery of probiotic bacteria, either as part of the natural microbiota or as added functional strains. Thermophilic fermented milks, characterized by lactate production and fermentation at 40–45 °C, include one of the most widely consumed products: yogurt. Yogurt is a viscous fermented product obtained from heat-treated milk through the activity of two homofermentative lactic acid bacteria species, namely *Streptococcus thermophilus* and *Lactobacillus delbrueckii* subsp. *bulgaricus*.

The proto cooperation between these two species is well documented and results in faster fermentation kinetics and a higher accumulation of compounds with significant sensory, textural, and nutritional impact. In some countries, legislation restricts the term “yogurt” to milk fermented exclusively by these two species, whereas in others the definition may be extended to fermented milk produced with *Streptococcus thermophilus* and *Lactobacillus delbrueckii* subsp. *bulgaricus*, even when additional probiotic microorganisms are present (6). Other products that have very similar characteristics to those just described for yogurt are Ayran (popular in Turkey and some Balkan and Caucasian countries) and Matzoon (popular in Armenia). Mesophilic fermented milks are produced through fermentations carried out at temperatures ranging from 20 to 30 °C. The process is primarily driven by LAB, including *Lactococcus lactis*, *Lactiplantibacillus plantarum*, *Limosilactobacillus fermentum*, *Lactobacillus helveticus*, *Streptococcus thermophilus*, and *Leuconostoc mesenteroides*. In addition to bacterial species, certain yeasts and filamentous fungi, such as *Geotrichum candidum*, *Kluyveromyces marxianus*, and *Pichia fermentans*, may also contribute to fermentation dynamics and product characteristics.

Kefir is one of the most important products classified within both acid and alcoholic fermentation processes, in which fermentation is carried out by complex microbial consortia organized in polysaccharide-rich granules. These granules contain lactic acid bacteria (LAB), including *Lactococcus lactis*, *Lactobacillus acidophilus*, *Lactobacillus kefiranofaciens*, and *Streptococcus thermophilus*, and yeasts (*K. marxianus, Torula kefir, Saccharomyces cerevisiae*) and other bacterial species. This consortium leads to the production of lactic acid and small amounts of ethanol and CO_2_, which confers a slight effervescence to the final product. Similar microbial characteristics are shown by kumis, typical of Central and East Asian steppes, obtained by mare milk and shubat (or chal) obtained from camel milk (18, 21).

Acidophilus milks are fermented milks obtained by adding *L. acidophilus* to milk, usually pasteurized and incubated at 37 °C. This species imparts a strong acidic taste that is not always pleasant. Therefore, its consumption is usually associated with consumers who seek the health and functional aspects that this species brings.

### 2.3 Cheese: diversity, classification, and microbial contributions

Given the wide variety of typologies, the definition of “cheese” is not simple and shows variations, even relevant ones, in the different national legislations. For this review, we use as a reference the definition given by WHO and FAO (22).

**Figure 1:**
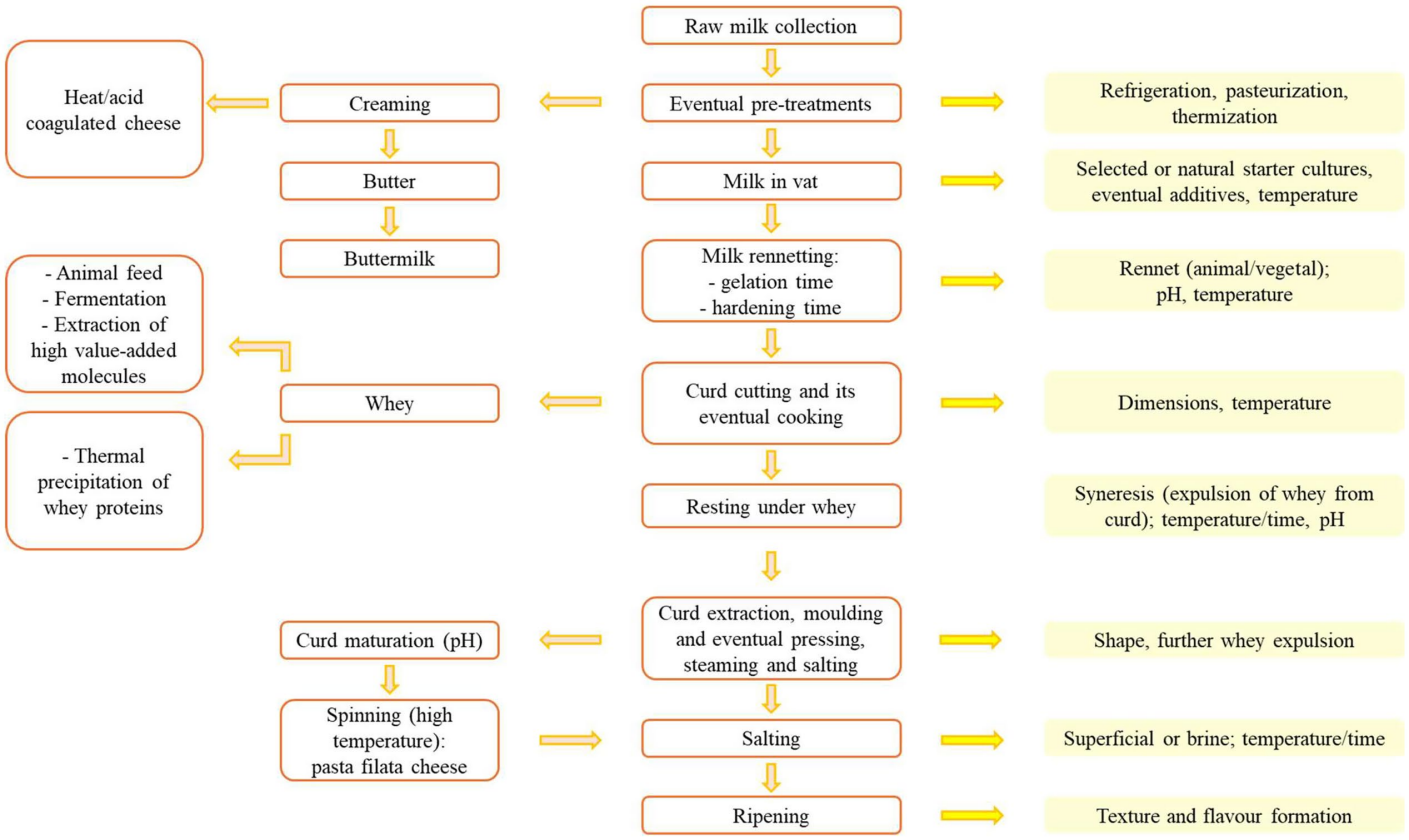
A general flowsheet for cheese production. In the yellow boxes, the main technological factors influencing each step are reported.

Cheese production is essentially a process in which the casein suspension of milk is destabilized through two distinct mechanisms that coexist, albeit in different ratios depending on the cheese considered (Figure 1).

The first mechanism is related to the reduction in pH resulting from microbial activity of selected or natural starter cultures, with effects similar to those observed in fermented milks, although acidification does not normally reach such low levels. The second destabilization factor is related to the presence of enzymes (traditionally of animal or plant origin) that act on the k-casein, which stabilizes the colloidal suspension, detaching its hydrophilic portion (casein macropeptide) while the hydrophobic portion (para-k-casein) remains anchored to the other caseins. The enzyme complex responsible for this process is rennet, a preparation mainly composed of proteolytic enzymes, which may also contain minor lipolytic activities depending on its origin. Specifically, the enzyme responsible for cleaving *κ*-casein is chymosin (23). The result is the formation of a hydrophobic protein gel that tends to expel the aqueous fraction (whey) along with the water-soluble substances it contains, by a factor of 6–12 depending on the type of cheese, while also fat is partially entrapped in the casein gel (24).

The casein gel (curd) is separated from the whey, which constitutes most of the raw material. The large amounts of residual whey find uses in obtaining other dairy products (such as Ricotta) or in animal feed. In recent decades, residual whey has been increasingly used to obtain products with high economic and nutritional added value, starting from peptides, or even as a base for fermented beverages. Cheesemaking is followed by curd extraction and moulding and by a salting phase and maturation, the duration of which depends on the type of product (2, 25).

The microbial contribution to cheese production is essential from at least two perspectives. First, as previously noted, microorganisms play a key role during the cheesemaking stage by contributing to casein destabilization. In addition, the presence and metabolic activity of microorganisms (primarily lactic acid bacteria (LAB), although other microbial groups may also play a significant role) are central to driving cheese ripening and influencing the biochemical and sensory changes that occur during storage (16).

All industrially made cheeses, and many artisanal products are nowadays obtained by the addition of starter cultures to initiate fermentation processes (26). Starter cultures used in cheesemaking may consist of selected strains grown in pure culture or of natural starter cultures, such as whey or milk starters, which comprise complex mixed microbial populations obtained through well-defined propagation procedures (27–29).

Broadly, starter cultures can be classified as thermophilic or mesophilic, depending on the temperatures applied during cheesemaking. Thermophilic cultures are employed in cheeses manufactured at temperatures ranging from 40 to 54 °C and are mainly composed of *Lactobacillus helveticus, Lactobacillus delbrueckii* subsp. *bulgaricus, Lactobacillus delbrueckii* subsp. *lactis*, and *Streptococcus thermophilus*. In contrast, mesophilic cultures are used in cheeses produced at lower temperatures (typically below 40 °C) and are primarily composed of *Lactococcus lactis* (30). These cultures remain dominant throughout the early stages of cheesemaking, and they already initiate other enzymatic activities that will later be reflected in the nutritional and sensory quality of the cheese. However, particularly in long-ripened cheeses, the progressive reduction in storage temperature and the concomitant decrease in water activity (a_w_), resulting from salting and moisture loss during ripening, promote the selection of distinct microbial populations, especially non-starter lactic acid bacteria (NSLAB). These microorganisms play a major role in driving structural modifications and in shaping the sensory and nutritional characteristics of the cheese, being active during cheese ripening.

NSLAB may originate from raw milk (particularly when it is not subjected to heat treatment before cheesemaking) or from environmental contamination associated with processing surfaces, equipment, and the dairy environment. Numerous mesophilic LAB can participate in this stage, with the most relevant species including *Lacticaseibacillus casei*, *Lacticaseibacillus paracasei*, *Lacticaseibacillus rhamnosus*, *Lactiplantibacillus plantarum*, and *Limosilactobacillus fermentum*, as well as *Leuconostoc mesenteroides* and *Leuconostoc lactis*.

Fungi also play a crucial role in specific cheese varieties. *Penicillium roqueforti* is responsible for internal colonization in blue-veined cheeses, whereas *Penicillium camemberti* and *Geotrichum candidum* are characteristic of surface-ripened cheeses. In smear-ripened cheeses, several Actinobacteria, including *Micrococcus*, *Arthrobacter*, and *Corynebacterium* spp., contribute to ripening. Furthermore, *Propionibacterium freudenreichii* is essential for the formation of large eyes in Swiss-type cheeses (30).

**Figure 2:**
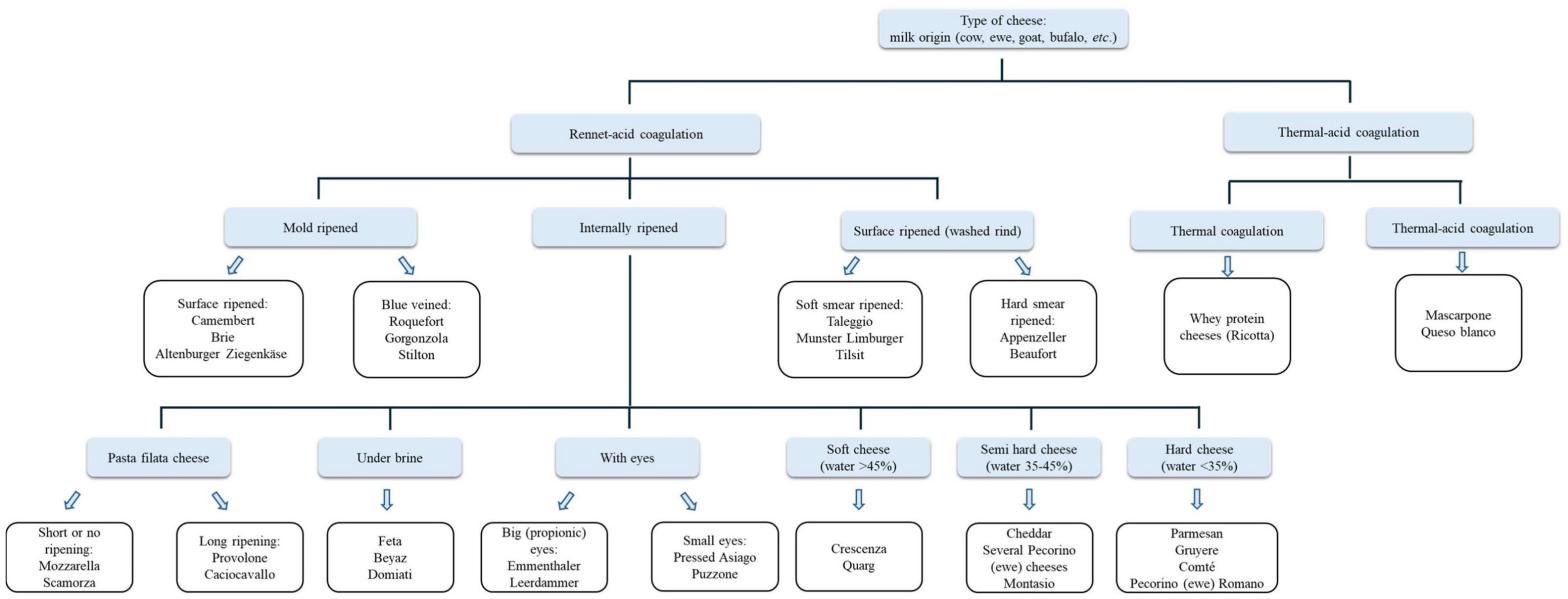
Classification of cheeses according to the characteristics of the final products and technological process adopted. For each group some examples are reported. Note that the same cheeses could be included in more than one category. Ricotta is included despite it cannot be considered a cheese according to WHO and FAO (22).

Due to different local traditions, a general classification of cheeses is not easy. Typically, they can be classified, in addition to the milk type used (cow, ewe, goat, water buffalo, *etc*.), according to the amount of residual water (from soft to very hard), the length of the ageing process, the temperature applied during cheesemaking, and the amount of residual fat. A possible classification is reported in Figure 2.

## 3 Health benefits of prebiotics, probiotics and postbiotics in dairy fermented foods

### 3.1 Probiotics and gut health

Probiotics are defined as “live microorganisms that, when consumed in sufficient amounts, provide a health benefit to the host” (31).

It has been shown that the gut microbiota plays a role in gut health and overall body health (32), that prebiotics contribute to human health by modulating the gut microbiota (33), that prebiotics and probiotics are present in fermented dairy products and that LAB in fermented dairy products contribute to gut health as probiotics (34), and that this contribution includes a ROS-reducing effect (35, 36). Therefore, fermented dairy products are promising as contributors to the reduction in the risk of many diseases, especially due to their probiotic and prebiotic-derived content.

Adaquate and balanced diet has been identified as a significant factor affecting the composition of the gut microbiota throughout the human lifespan. Dysbiosis, or an imbalance in the microbiota composition, has been linked to the onset of various chronic conditions. One non-pharmacological approach to promoting microbial balance is to consume fermented milk products rich in live microorganisms (37, 38).

Fermented milk products include beneficial probiotics (such as *Lacticaseibacillus rhamnosus* GG (LGG), *Bifidobacterium animalis subsp. lactis* BB12, *Lactiplantibacillus plantarum* WCFS1), prebiotics, the presence of beneficial bioactive peptides, vitamins, and other elements generated by bacteria. Fermented milk products have beneficial effects on gastrointestinal health and immune system balance. It can modulate the inflammatory diseases and also it is potential to improve the health of the body in a range of gastrointestinal disorders, such as diarrhea, Irritable Bowel Syndrome, bloating.

In the context of dairy-based fermented foods, LAB and *Bifidobacterium* are among the most frequently studied genera in probiotic research. However, it is important to distinguish between starter cultures used in fermentation and specific probiotic strains, as probiotic effects are strain-dependent and not all starter cultures confer documented health benefits (39, 40). These organisms are provided within a protective milk matrix, which has been shown to enhance their viability during gastrointestinal transit. The structural composition of coagulum and milk proteins has been shown to facilitate the encapsulation and protection of probiotic cells, thereby promoting their survival under conditions of gastric and duodenal stress and promoting their functional delivery to the intestine (39).

From a mechanistic perspective, probiotics contribute to gut homeostasis via three distinct mechanisms: Modulation of epithelial barrier function, competitive exclusion of pathogens, and immunomodulation. Firstly, it has been demonstrated that several strains (*Lactiplantibacillus plantarum* 299v, *Lacticaseibacillus rhamnosus* GG etc.) have the capacity to upregulate tight-junction and mucin genes (e.g., MUC2/MUC3) and stabilise monolayer resistance. The outcomes of this process have been shown to result in a strengthening of barrier integrity and a limitation of pathogen adherence (41–44). Secondly, the presence of probiotic metabolites and competition for colonisation sites has been indicated as a factor in the reduction of colonization and the expression of pathogenic effects within the gut microbiota (40). Thirdly, probiotics have been shown to modulate innate and adaptive immune responses by regulating pattern-recognition and inflammatory signalling. A substantial body of evidence indicates that certain strains (such as *Bifidobacterium lactis* strain BB12, *Lactobacillus plantarum* 2,142, *Saccharomyces cerevisiae* strain CNCM I-3856) may influence Toll-like receptor (TLR) and NOD-like receptor pathways, suppress NF-κB activation, and modify cytokine profiles towards anti-inflammatory states in epithelial cells, antigen-presenting cells, and macrophages (44). *In vitro* models have shown that probiotic strains (*Lactococcus lactis* subsp. *cremoris* FC, *Bifidobacterium lactis* NCC362, *Bifidobacterium longum* NCC2705, etc.) suppress NF-κB nuclear translocation (45, 46). In intestinal and immune cell systems, probiotics also modulate the expression of IL-8, IL-6, TNF-*α*, and TLR2/3/4/9, supporting their roles in microbiota regulation, epithelial barrier enhancement, and mucosal immune modulation (44).

It has been shown that starter bacteria from yogurt can survive passage through the gastrointestinal tract (39, 47). In addition to basic survival considerations, population-level evidence has found an association between the consumption of fermented milk and yogurt and improved metabolic health, as indicated by factors such as glycemic and lipid profiles (40). However, the heterogeneity of the data and the potential for confounding factors necessitate cautious interpretation of these results and further trials to support stronger conclusions. Clinical and experimental evidence highlights the importance of strain specificity and dosage thresholds (approximately 10^8^–10^9^ CFU/day) for consistent probiotic benefits, with recommendations that fermented products maintain a viability of at least 10^6^ CFU/g at the end of their shelf life (40).

In addition to viability, the dairy matrix plays a critical role in determining probiotic functionality. Coagulated proteins have the capacity to encapsulate cells, and the use of microencapsulation and technological strategies (e.g., Exopolysaccharides (EPS)-producing strains, pH/oxygen control, addition of prebiotics) may further improve survival through manufacturing, storage, and digestion (39, 40). While some fermented milks contain complex microbial consortia with the capacity to modulate the microbiota extensively (48), the precise causal pathways linking community shifts to clinical outcomes are still being studied. This emphasises the necessity for well-controlled human studies that address strain, dose, matrix, and host factors (44, 48).

#### 3.1.1 Signaling pathways

Metabolites in fermented dairy products have been shown *in vitro* and in animal models to modulate inflammatory pathways implicated in inflammatory bowel disease by modulating important proinflammatory cytokines, such as ERK 1/2, JNK, p38, p50, and p65, as well as MAPK and NF-κβ signaling pathways (49).

In a study using Caco-2 intestinal epithelial cells (*in vitro*) and a dextran sulfate sodium (DSS)-induced ulcerative colitis mouse model (*in vivo*), *Lacticaseibacillus rhamnosus* 1.0320 and its heat-killed postbiotic form were reported to alleviate colitis by modulating gut microbiota and intestinal metabolism, significantly reducing ulcerative colitis symptoms, colon damage, oxidative stress, and myeloperoxidase (MPO) activity. Mechanistically, they suppressed key proteins of the TLR4/MAPK/NF-κB signaling pathway (TLR4, MyD88, JNK, p38, and p65), decreased pro-inflammatory cytokine secretion, and increased short-chain fatty acid (SCFA) production (50). A review study has reported that probiotics in fermented products, such as yogurt and kefir reduce inflammation by decreasing immunogenic dendritic cells (DC) activation on intestinal epithelial cells, thereby supporting tolerance formation (51).

#### 3.1.2 Safety of probiotics

Probiotics have been reported to have beneficial effects in maintaining intestinal homeostasis and barrier integrity. They affect the intestinal barrier by preventing intestinal barrier dysfunction, inhibiting bacterial translocation and adhesion, and increasing the expression of tight-junction proteins. Furthermore, probiotics modulate the pH of the intestinal lumen by releasing bioactive peptides, lactic acid, and acetic acid, which have antimicrobial effects. This inhibits the growth of non-comensal pathogenic microorganisms and the production of bacterial toxins. Fermented dairy products containing probiotics can generally be safely consumed by healthy individuals; however, in critically ill, postoperative, or immunocompromised patients, caution is advised due to potential side effects (52, 53). In addition, they modulate the host’s immune system, inhibiting autoaggressive reactions. However, probiotics can cause side effects such as headaches, digestive discomfort, constipation, and unwanted weight loss (54). The FAO/WHO guidelines classify potential side effects associated with probiotics into four main categories: adverse metabolic effects, systemic infections, transmission of antibiotic resistance genes, and excessive immune stimulation (53).

Probiotics can be added to foods in the form of fermented dairy products or non-dairy foods, or consumed as supplements (55). In the food industry, probiotics can be found in many products such as yogurt, frozen fermented milk desserts, ice cream, cheese and cheese products, fruit juices, baby foods, and cereals (55, 56). The sensitivity of probiotics to heat treatment and factors such as gastrointestinal stress in the human body create limitations on their use in the food industry (57).

Fermented dairy products containing probiotics are generally well tolerated and safe for healthy individuals, with mild side effects such as diarrhea or constipation rarely occurring (58, 59).

Although probiotics have proven benefits for gastrointestinal disorders, evidence for their effects on non-gastrointestinal conditions, such as cancer or depression, remains limited (60–62). Therefore, patients—especially those in high-risk categories—should consult a healthcare professional before use, and safety monitoring is recommended during long-term consumption (22, 53).

### 3.2 Immune system modulation

Maintaining the balance of the gut microbiota is very important (63). Because, the gut microbiota provides various nutrients for host health, modulates immune system responses, regulates energy balance, and supports the defense system against pathogens (64–66). It is known that probiotics, when taken in appropriate doses, contribute significantly to maintaining a healthy microbiota (54, 67).

Probiotics can modulate Toll-like receptors (TLRs) expressed on immune system cells and influence downstream signaling pathways such as nuclear factor kappa B (NF-κB) and mitogen-activated protein kinases (MAPKs) (68). Depending on the host–microbe interaction context, innate immune responses may be differentially regulated, which may result in either immune activation or modulation of pro-inflammatory cytokine and chemokine levels (69). In an *in vitro* study, four different probiotic strains (*Lacticaseibacillus rhamnosus* GG, *Lactobacillus helveticus* IMAU70129, *Lacticaseibacillus casei* IMAU60214, and *Lacticaseibacillus rhamnosus* KLSD) were reported to stimulate innate immune responses in human macrophages by enhancing phagocytic activity and activating TLR2 and NF-κB pp65 signaling pathways. The observed increase in ROS production was suggested to function as part of the macrophage antimicrobial defense response rather than as a detrimental oxidative effect (70). In a mouse model study conducted by Kwon et al. (71), a probiotic mixture (*Lactobacillus acidophilus*, *Limosilactobacillus reuteri*, *L. casei*, *Bifidobacterium bifidum*, and *Streptococcus thermophilus*) was administered, resulting in an increase in CD4 + Foxp3 + regulatory T cells (Treg) and a decrease in T helper (Th) cell numbers (71). It has been suggested that this effect may help prevent the progression of atopic dermatitis, rheumatoid arthritis, and inflammatory bowel disease, and may contribute to the regulation of immune system dysfunction associated with these diseases (71). In addition, in a study using *in vitro* and *in vivo* methods it has been reported that the expression of the transcription factor Foxp3 increased and Tregs were induced in infant mice (0–6 weeks) administered *B. longum* AH1206, and that this could have a protective effect against respiratory allergy (72).

Various studies have also shown that prebiotics and postbiotics-metabolites derived from probiotics-maintain the homeostasis of the gut microbiota, modulate immune system responses, protect intestinal barrier integrity, and demonstrate beneficial effects against numerous chronic disorders such as Type 2 Diabetes Mellitus, cancer, obesity, cardiovascular diseases, and central nervous system disorders (73–77).

The consumption of fermented dairy products plays an important role in enriching the prebiotic population (78). Prebiotics were defined in 2008 by the International Scientific Association of Probiotics and Prebiotics (ISAPP) as “a selective fermentable component that induces specific changes in the composition and/or activity of the gastrointestinal microbiota, thereby conferring a benefit or benefits to the host” (79). Prebiotics present in fermented dairy products, particularly galacto-oligosaccharides and inulin-type fructans, can regulate immune responses by activating natural killer (NK) cells as well as B and T lymphocytes, suppressing the expression of proinflammatory cytokines, and promoting anti-inflammatory cytokine production (80). In particular, inulin and oligofructose have been reported to modulate both the systemic immune system and the local intestinal immune response (81).

Postbiotics regulate the expression of CD8^+^T cells, interferon and can affect the immune system responses (82). Tryptophan metabolites can inhibit inflammation by affecting T cell aromatic receptors (83). In addition, postbiotics can influence the immune system response by modulating inflammatory cytokines. In a study, it was noted that *Lacticaseibacillus rhamnosus* GG effector protein HM0539, a postbiotic, exhibits immunomodulatory effects. It has been reported that it inhibits expression of inducible nitric oxide synthase (iNOS) and cyclooxygenase-2 (COX-2) in the *in vitro* cell cultures and *in vivo* mouse colitis models, and suppresses nitric oxide (NO) and prostaglandin (PG)E2 production via the TLR4/MyD88/NF-κB signaling pathways (84). In an *ex vivo* culture model obtained from patients with diarrhea, it was found that the postbiotic cell-free supernatant from *Lacticaseibacillus rhamnosus* DG reduced the expression levels of IL-6, IL-1α, and IL-8, while increasing the expression level of IL-10, thereby potentially reducing the pro-inflammatory immune system response and increasing the anti-inflammatory immune system response (85).

**Figure 3:**
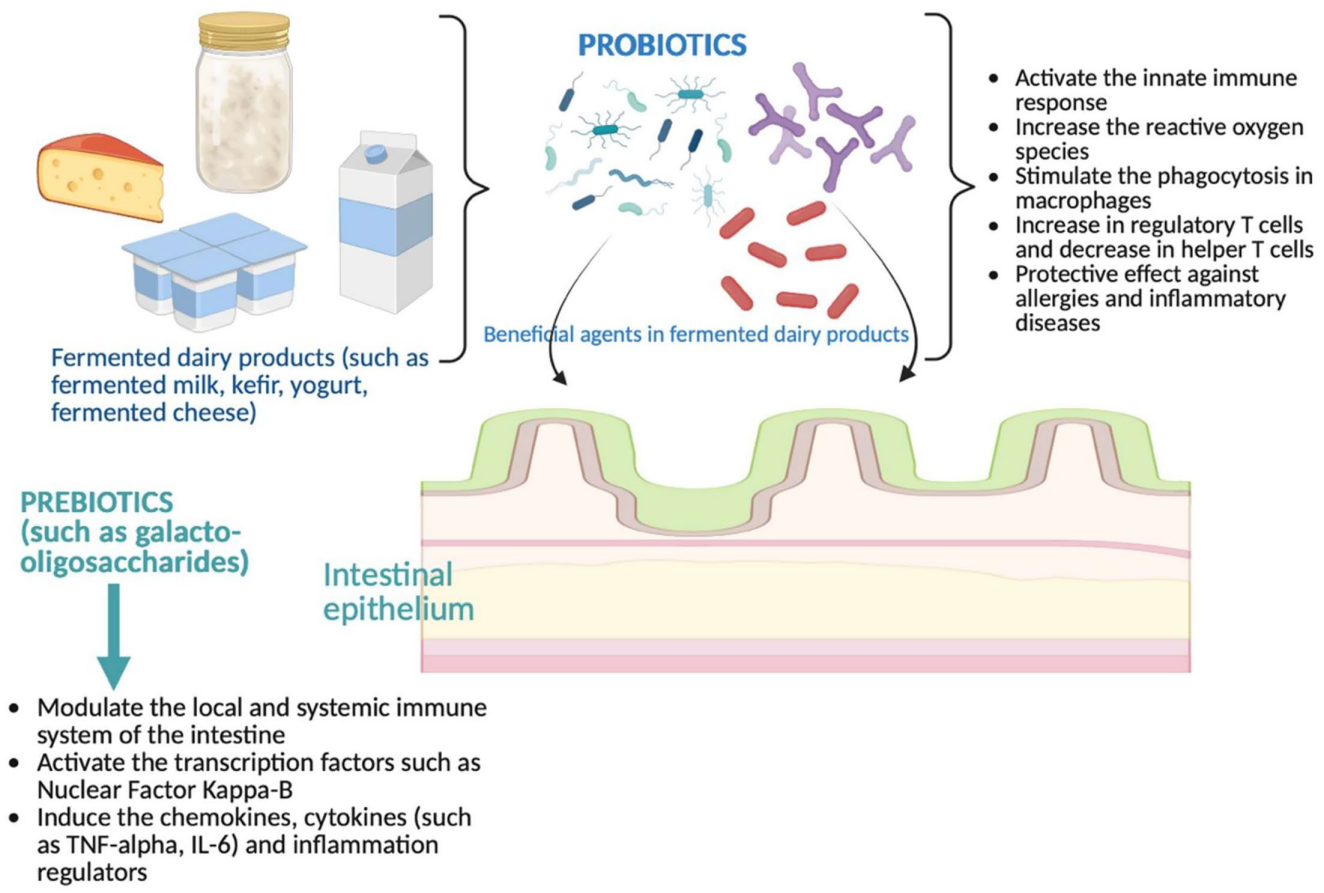
Pharmacological effects of prebiotics and probiotics in fermented dairy products. TNF: Tumor necrosis factor, Interleukin: IL (Created with BioRender.com).

The criterion for the immunomodulatory capacity of fermented milk products is that they exhibit significantly different immunomodulatory profiles (86). Fermented milk products must contain a minimum number of live microorganisms (more than 10^8^ CFU/g) at the time of consumption. For example, both LAB and *P. freudenreichii* have high immunostimulatory potential in terms of their probiotic properties. The immunomodulatory capacity of fermented dairy products increases to the extent that their ecosystem consists of different bacterial species that can exhibit different immunological properties, and a synergistic effect may occur. After growth, the surface proteome may differ significantly, which may also cause changes in immunomodulatory properties (86) (Figure 3).

### 3.3 Antimicrobial effects

During milk fermentation, LAB converts lactose into lactic acid, lowering the pH of the environment and inhibiting the growth and proliferation of microorganisms other than LAB. Additionally, LAB can synthesize certain antimicrobial components such as reuterin, bacteriocin, and diacetyl (87). Bacteriocin has been reported to exhibit bacteriostatic or bactericidal effects (88). Furthermore, it has been noted that LAB produces antimicrobial components such as ethanol, organic acids, and hydrogen peroxide (89). It has been documented that specific peptides (Antimicrobial peptides such as isracidin, k-casecidin, lactoferricin; metal-binding peptides such as casein phosphopeptides; and certain milk-derived peptides reported to modulate cell cycle and apoptosis) are released during LAB proteolysis, and these peptides may have potential pharmacological effects such as antioxidant, antimicrobial, anticancer, and antifungal properties (12).

Kefir, a fermented milk product, has been reported to have a wide range of pharmacological activities, including anti-allergic, anti-carcinogenic, wound-healing, immunomodulatory, and antimicrobial effects (90). Several studies have reported that kefir and its exopolysaccharide component, kefiran, exhibit antibacterial and antifungal activities against various pathogens; however, these antimicrobial effects are not considered comparable to those of conventional antimicrobial agents (90–93). *Lactobacillus* species (*Lactobacillus acidophilus* and *Lactobacillus kefiranofaciens*) obtained from kefir have been reported to exhibit antimicrobial activity against various pathogens such as *Staphylococcus aureus*, *Escherichia* (*E.*) *coli*, *Salmonella typhimurium*, *Yersinia enterocolitica*, and *Pseudomonas aeruginosa* (90).

It has been reported that acetic acid produced by LAB penetrates the cell membranes of fungi, thereby exerting an antifungal effect (94). It has been observed that phenyllactic acid (PLA), which is produced from the amino acid phenylalanine released during the proteolysis of milk proteins (such as casein and whey proteins) in fermented dairy products, exhibits antifungal activity by interfering with energy metabolism and fungal cell membranes (94, 95). Additionally, it has been discovered that organic acids including lactic acid, propionic acid, and formic acid contribute to antifungal action. Propionic and formic acids are shown to be beneficial by infiltrating fungal cells and upsetting their metabolic processes and pH balance (94)(Table 1).

**Table 1:** Antimicrobial effects of fermented dairy products.

Probiotic	Fermented dairy products	Mechanism of antimicrobial effect	Target pathogens	References
*Lactobacillus acidophilus*	Yogurt, kefir, probiotic-containing fermented dairy products	Production of bacteriocins and acidic metabolites (organic acids such as lactic and citric acids)	*Bacillus subtilis, C. perfringens*, *Candida* spp.*, E. coli*, *Staphylococcus aureus*	(208, 209)
*Lactococcus lactis*	Fermented cheese, fermented butter, and other fermented dairy products	H₂O₂, lactic acid, bacteriocin, nisin production	*Staphylococcus aureus, E. coli*	(210–212)
*Limosilactobacillus reuteri*	Fermented milk and dairy products (such as yogurt, kefir)	Reuterin production	*E. coli*, *Staphylococcus aureus, Listeria monocytogenes, Salmonella enterica and Salmonella* spp.	(210, 213)
*Bifidobacterium* spp.	Fermented milk	Acetate, bacteriocin, H_2_O_2_, organic acid production, lipoprotein A found in the cell wall	*E. coli, Pseudomonas aeruginosa, Enterococcus faecalis, Salmonella typhi, Streptococcus mutans, Listeria monocytogenes*	(214–216)
Kefir culture (lactic acid and acetic acid bacteria)	Kefir	Polysaccharides (exopolysaccharides), bacteriocin, diacetyl, organic acids, ethyl alcohol, hydrogen peroxides production	*E. coli*, *Staphylococcus aureus, Listeria monocytogenes*, *Salmonella enteritidis, Bacillus cereus, Pseudomonas aeruginosa, Cronobacter sakazakii*	(217)
Yoghurt starter cultures (*L. delbrueckii subsp. bulgaricus*, *S. thermophilus*)	Yoghurt, fermented milk	Production of bacteriocins, peptides, and organic acids (such as lactic acid, citric, acetic, succinic, and butyric acids)	*E. coli, Listeria monocytogenes, Klebsiella pneumoniae, Salmonella typhimurium, Helicobacter pylori,*	(100, 218–222)

C, Clostridium; E, Escherichia; H_2_O_2_, Hydrogen peroxide; L, Lactobacillus; S, Streptococcus.

### 3.4 Digestive and cardiometabolic effects

#### 3.4.1 Lactose intolerance

Lactose intolerance is a metabolic disorder caused by the inability to digest lactose due to insufficient levels of the lactase (*β*-galactosidase) enzyme. Lactose intolerance can be divided into three groups. Primary lactose intolerance is genetically determined and is usually associated with low lactase levels after breastfeeding. Secondary lactose intolerance usually occurs when the intestinal mucosal surface is damaged due to disease, surgery, radiation, or medications. Secondary lactose intolerance can cause mucosal diseases such as celiac disease and Crohn’s disease. Congenital lactose intolerance, on the other hand, is extremely rare and occurs only when the lactase enzyme is completely absent (96). One of the first recommended methods for improving lactose intolerance symptoms is the use of exogenous lactase enzyme. When taken before consuming foods containing lactose, this enzyme can reduce the possibility of intolerance symptoms occurring and even alleviate symptoms. However, due to the possibility of consuming more lactose than the enzyme can digest, this method may not always meet the individual’s needs. Recommended alternatives include modulating the gut microbiota through the consumption of probiotics and prebiotics. In addition to contributing to overall health, probiotics and prebiotics also help alleviate lactose intolerance symptoms by promoting the growth of microorganisms that digest lactose by its own lactase enzyme (97, 98). Although some studies have investigated the effects of probiotics on primary lactose intolerance, the available evidence remains limited and inconsistent, particularly regarding secondary lactose intolerance; therefore, further well-designed studies are needed (97, 99).

Yogurt, a fermented dairy product, has been reported to enrich the gut microbiota in favor of beneficial microbial communities and may be beneficial for gastrointestinal disorders such as lactose intolerance, inflammatory bowel disease, and irritable bowel syndrome (100, 101). Fermentation of dairy products increases their nutritional value and bioavailability, and certain lactic acid bacteria (LAB) strains can reduce lactose intolerance. LAB reduce lactose content through their lactase activity and contribute to lactose digestion when consumed by lactose-intolerant individuals (102–104).

It has been reported that kefir may provide beneficial effects in individuals with lactose intolerance by facilitating lactose digestion and improving its tolerance, thereby supporting gastrointestinal function (105). It has been stated that all cheeses, except fresh cheese and soft cheese, can be consumed by individuals with lactose intolerance because they do not contain lactose or are low in lactose (106). On the other hand, many studies have shown that people with lactose intolerance can consume acidophilus milk (containing *Lactobacillus acidophilus*) (107).

The impact of yogurt enhanced with *Bifidobacterium* sp. and *Lactobacillus acidophilus* on patients with lactose intolerance was examined in a study by Masoumi et al. two groups of 55 lactose intolerant patients (39 females, 16 males) were randomly assigned. For a week, the first group consume yogurt devoid of probiotics, and the second group consume probiotic yogurt that contained *Bifidobacterium* sp. and *Lactobacillus acidophilus*. According to the study’s findings, patients who consume probiotic-rich yogurt experienced fewer symptoms of lactose intolerance, including cramping, gas, bloating, and stomach pain, than those in the control group (101). In a clinical study performed by Hertzler and Clancy, 15 healthy adults (7 female, 8 male) with lactose intolerance consumed milk (non-fermented control), plain yogurt, plain kefir, flavored yogurt, and flavored kefir containing lactose in separate treatments (407 g, 378 g, 508 g, 428 g, and 519 g, respectively), and lactose intolerance symptoms were assessed. Compared with milk, yogurt and kefir containing probiotic cultures were associated with markedly lower gastrointestinal symptom severity in individuals with lactose maldigestion. Specifically, yogurt and kefir reduced the perceived severity of flatulence by approximately 54–71% relative to milk, while abdominal pain and diarrhea symptoms remained minimal across treatments (108).

Galacto-oligosaccharides, which are prebiotics, are indigestible carbohydrates composed of a galactose molecule and a saccharide molecule (109). The effects of probiotic *Bifidobacterium* species and prebiotic galacto-oligosaccharides on lactose intolerance have been investigated in numerous recent clinical studies. They also facilitate digestion by breaking down lactose through the beta-galactosidase enzyme they contain, while galacto-oligosaccharides have been reported to enhance lactose metabolism by nourishing beneficial bacteria in the intestinal microbiota (110–112).

#### 3.4.2 Antihypertensive bioactive peptides (ACE-inhibitors)

Fermented dairy products have also been reported to exhibit antihypertensive effects due to the bioactive peptides they contain (113). It has been noted that the bioactive peptides in fermented dairy products are formed as a result of microbial proteolysis and the enzymatic hydrolysis of milk proteins, particularly whey protein and casein. During the fermentation process, LAB secrete protease enzymes that release free tripeptides such as Isoleucine-Proline-Proline (Ile-Pro-Pro) (IPP) and Valine-Proline-Proline (Val-Pro-Pro) (VPP) from beta and kappa-casein (114, 115). Although these peptides are suggested to inhibit ACE at low micromolar concentrations (often reported with IC50 values below 100 μM in potent fractions), their potency and clinical efficacy have not yet been elucidated. However, they cannot be compared to the potency and clinical efficacy of ACE inhibitors (114, 115). Studies on fermented dairy products enriched with the bioactive peptides IPP and VPP have demonstrated reductions in systolic blood pressure (mean decreases ranging from 1.3 to 5.6 mmHg) in individuals with prehypertension or hypertension (114, 116). Numerous studies have demonstrated the ACE-inhibitory properties of various cheese varieties, which have been attributed to specific bioactive peptides, including *β*-casein-derived peptides such as β-casomorphin-7 and αs1-casein-derived peptides such as αs1-casokinin-6 and αs1-casokinin-5 (117, 118). It has been reported that the ACE-inhibitory potential of αs1-casein-derived bioactive oligopeptides, such as αs1-casokinin-6 and αs1-casokinin-5, which originate from the enzymatic hydrolysis of milk casein, is lower than that of the more potent tripeptides VPP and IPP (12).

#### 3.4.3 Antioxidant bioactive peptides

Kefir, a fermented milk product, has been evaluated for its antioxidant potential in various scientific studies (119). In an *in vitro* study conducted by Güven et al. (120), milk-derived kefir was evaluated under an oxidative stress model, where lipid peroxidation was assessed by measuring malondialdehyde (MDA) levels, along with reduced glutathione (GSH) content and the activities of antioxidant enzymes including glutathione peroxidase, glutathione S-transferase, and catalase; kefir showed stronger antioxidant activity than vitamin E based on its effects in this system. In another study by Liu et al. (121), kefir made from cow’s and goat’s milk was reported to have the potential to bind superoxide and 1,1-diphenyl-2-picrylhydrazyl (DPPH) radicals. Another fermented milk beverage, koumiss, has been found to modulate oxidative stress markers, decrease glutathione levels, and increase antioxidant capacity (122). The antioxidant activity of bioactive peptides (such as casein-derived peptides, whey protein-derived peptides) found in Cheddar cheese, a fermented dairy product, has been investigated in several studies. Silva et al. (123) reported that peptides generated during cheese ripening exhibited significant free radical scavenging activity, while Pritchard et al. (124) demonstrated that cheese-derived peptides showed antioxidant capacity through inhibition of oxidative reactions and enhancement of radical scavenging mechanisms (123, 124). In an *in vitro* study, the antioxidant capacity of cheddar cheese produced with and without starter cultures at different stages of maturation was evaluated. The results showed that antioxidant activity was dependent on the ripening stage, and that cheddar cheese produced with starter cultures had higher 2,2′-azino-bis(3-ethylbenzothiazoline-6-sulfonic acid) (ABTS) radical scavenging activity. Trolox equivalent antioxidant capacity also increased throughout the ripening process (125). In another *in vitro* study conducted by Huma et al. (126), water-soluble peptides derived from Cheddar cheese, including casein-derived peptides (β-casein and αs1-casein fragments) and whey protein-derived peptides enriched in hydrophobic and proline-containing sequences, were shown to exhibit antioxidant activity in Caco-2 intestinal epithelial cell culture models by modulating oxidative stress markers (Table 2).

**Table 2:** Bioactive peptides in fermented dairy products and their pharmacological effects.

Bioactive peptide	Fermented dairy products	Pharmacological effect	References
IPP & VPP (Lactotripeptides)	Fermented milk, yogurt, kefir, fermented cheese	Antihypertensive effect (with ACE inhibition)	(115, 114, 210)
αs1-casein (24–32) & β-casein (193–209)	Fermented milk, yogurt, kefir, fermented cheese	ACE inhibition, opioid-like activity	(210, 223)
Lactoferricin	Fermented milk	Antioxidant, immunomodulatory, inhibition of pathogenic bacterial growth through disruption of bacterial cell membrane integrity	(210, 224)
κ-Casein fragments	Yogurt, fermented milk	Immunomodulator (Increased IL 10, T-reg activation, reduction of inflammatory response)	(210)
LGG-derived soluble proteins (p40 and p75)	*Lacticaseibacillus rhamnosus* GG fermented milk	Caco-2 tight junction integrity preservation	(210, 225, 226)
Postbiotic peptides, small molecules similar to GABA	Yogurt, kefir	Anxiolytic effect	(210)
SCFA (Acetate, Propionate, Butyrate)	Yogurt	Improvement of serum and liver lipid profile, hypoglycemic effect, improvement of insulin resistance	(218)

IPP, Isoleucine-Proline-Proline; VPP, Valine-Proline-Proline; ACE, Angiotensin I Converting Enzyme; IL-10, Interleukin 10; T-reg, Regulatory T cells; GABA, γ-aminobutyric acid; SCFA, Short Chain Fatty Acids.

#### 3.4.4 Cholesterol management

**Figure 4:**
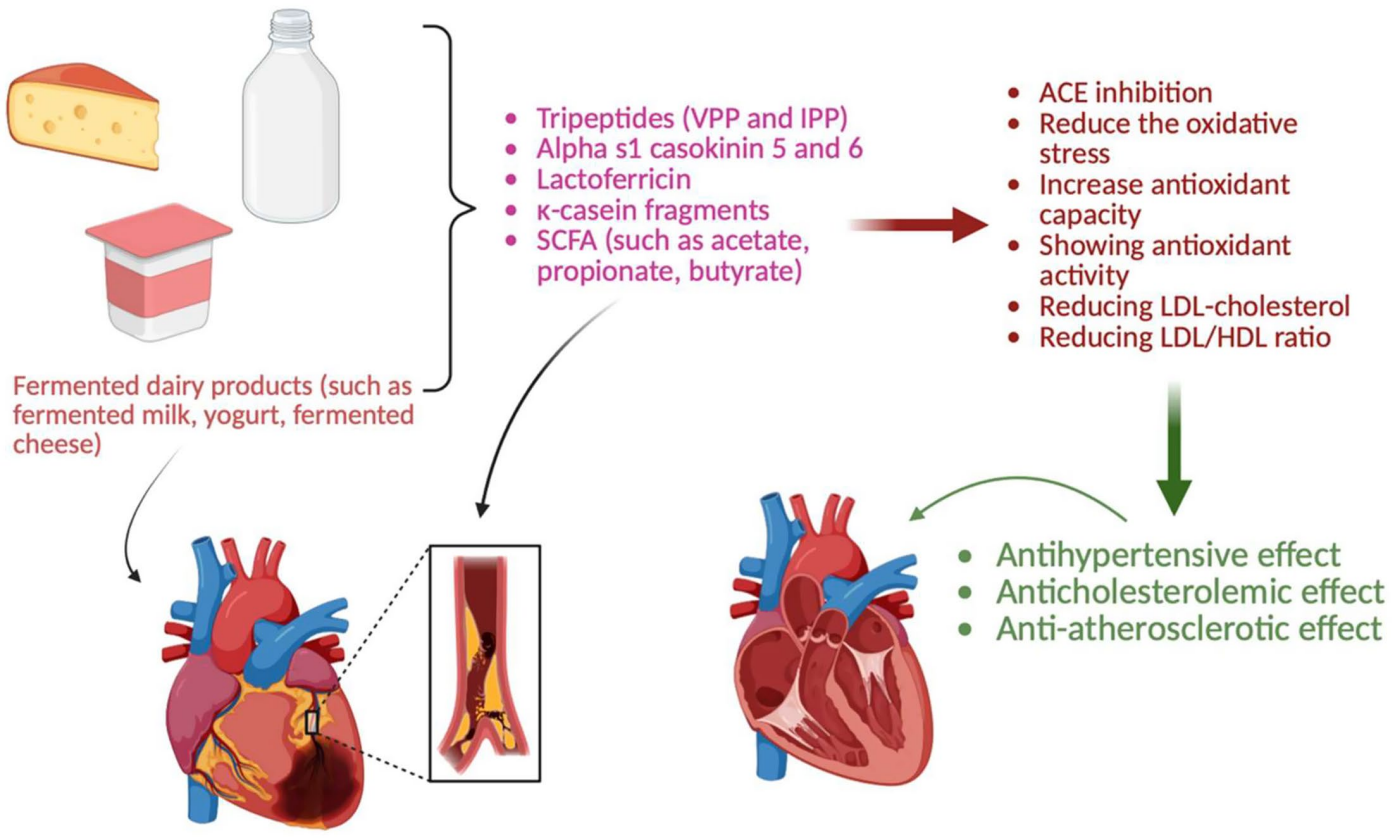
Cardiovascular effects of fermented dairy products. IPP: Isoleucine-Proline-Proline, VPP: Valine-Proline-Proline, SCFA: Short chain fatty acids, ACE: Angiotensin I converting enzyme, LDL: Low density lipoprotein, HDL: High density lipoprotein (Created with BioRender.com).

Some studies have shown a significant relationship between total and Low Density Lipoprotein (LDL) cholesterol and the risk of cardiovascular disease (127–129). Concerns regarding conventional lipid-lowering therapies, including potential adverse effects such as muscle-related symptoms, liver enzyme alterations, drug intolerance, and long-term safety issues, have led to growing interest in complementary treatments to improve lipid profiles (130). Fermented dairy products are widely recognized as probiotic foods with considerable potential health benefits (131). A systematic review and meta-analysis of 39 randomized controlled trials including a total of n = 2,237 participants reported that consumption of probiotic fermented milk products significantly reduced total cholesterol and LDL cholesterol levels. The pooled effect sizes indicated reductions in LDL cholesterol (Weighted Mean Difference (WMD): −7.34 mg/dL; 95% Confidence Interval (CI): −10.04 to −4.65; *p <* 0.001) and total cholesterol (WMD: −8.30 mg/dL; 95% CI: −11.42 to −5.18; *p <* 0.001) (129). In a randomized controlled trial involving 29 individuals with baseline LDL cholesterol levels >130 mg/dL, consumption of kefir resulted in a 7.6% reduction in LDL cholesterol and a 5.4% decrease in apolipoprotein B (apoB) levels, whereas no comparable changes were observed in participants consuming unfermented milk (132). In a controlled study, it was reported that in those who consumed yogurt that contained strains of *Bifidobacterium lactis* Bb12 and *Lactobacillus acidophilus* La5, LDL cholesterol was reduced by 7.45%, total cholesterol was reduced by 4.54% and the LDL/High Density Lipoprotein (HDL) ratio was also reduced compared to those who consumed plain yogurt; however, HDL cholesterol and triglyceride changes were not significant (133). In another clinical study, blood cholesterol and triglyceride levels decreased in 16 hyperlipidemic individuals who consumed koumiss (350 mL/day for 15 days) (134). A study using an *in vivo* hyperlipidemic rat model reported that a probiotic strain (*Lactobacillus casei* Zhang) isolated from koumiss reduces hyperlipidemia (135) (Figure 4).

### 3.5 Bone health and nutrient bioavailability

Bone health is affected by various intrinsic and extrinsic factors, including genetics, nutrients and environmental factors. Inadequate nutrient intake negatively affects bone mineralization, especially during childhood and adolescence, and is associated with lower bone mass and increased fracture risk in later life (136, 137). For instance, fermented dairy products such as yogurt, kefir and some fermented cheeses have been shown to promote balanced bone mineralization by increasing the bioavailability of minerals such as calcium, an important regulator of bone metabolism. Both clinical and experimental studies show that bioactive compounds present in fermented dairy products, such as kefir, yogurt, and fermented cheese varieties, contribute to bone health by enhancing osteoblast activity, inhibiting bone resorption, and improving bone microstructure (138–140).

Milk and dairy products, especially fermented types, are rich sources of important nutrients that support bone health. They play a role in skeletal system development and endurance with their contents such as calcium, phosphorus, magnesium, potassium, vitamin B12, vitamin D, vitamin K and protein (105). It has been reported that fermented dairy products containing *Lactobacillus* spp. increase calcium absorption and support bone health through various biological mechanisms (141). *Lactobacilli* in fermented dairy products lower the pH of the intestinal environment after consumption, increasing the solubility of calcium ions, which facilitates absorption. In addition, casein phosphopeptides formed during the fermentation process form a complex with calcium, supporting its solubility and bioavailability (142). Calcium from these products has higher bioavailability compared to plants or nutritional supplements (136). Fermented dairy products such as yogurt, kefir and cheese not only provide these essential nutrients, but also acquire functional properties through fermentation. For example, lactic acid from the degradation of lactose increases the solubility of calcium by providing a more acidic environment in the intestine. Furthermore, specific peptides produced by certain bacterial strains during fermentation (such as casein phosphopeptides, CPPs) and the resulting decrease in intestinal pH play a supportive role in the absorption of calcium and other minerals in the gut. These peptides have been shown in both *in vitro* and *in vivo* studies to chelate calcium efficiently, enhance mineral uptake and osteoblast mineralization activity (136, 137, 140, 143).

Some strains of *Lactobacillus* spp. (e.g., *L. plantarum, L. delbrueckii*) contribute to intestinal transport of calcium by increasing the expression of transcellular transport proteins such as Transient Receptor Potential Vanilloid 6 (TRPV6), and intercellular junction proteins involved in paracellular absorption such as claudin-2 (Cld-2) (144).

CPPs increase calcium solubility in the lumen and upregulate the TRPV6 calcium channel in epithelial cells, improving bone mineralization (138, 140, 145). Fermentation releases more CPPs from casein via bacterial proteases, supporting this dual mechanism (146).

Furthermore, some specific peptides, such as *β*-casein (16–40), have been shown to increase calcium transport by upregulating the TRPV6, increasing calcium transit in human colon carcinoma cell line (Caco-2) and both supporting bone formation and preventing bone destruction (138, 147). In addition, it has been reported that some peptides obtained from milk proteins exhibit antioxidant properties; thus, they can support bone health by suppressing increased osteoclast activity due to oxidative stress and increasing osteoblast differentiation (138, 148). In a study, it was reported that kefir-fermented peptide-1 (KFP-1) isolated from kefir increased intestinal calcium absorption through TRPV6 channel; in addition, it suppressed osteoclastogenesis, the formation of cells responsible for bone destruction, and promoted osteoblastogenesis, the formation of cells responsible for bone formation, thus showing a significant osteoprotective effect in osteoporosis model (female ICR mice, 5 weeks old) (149). These findings suggest that bioactive peptides from fermented dairy products have a significant potential on bone health. It also prevents soft tissue calcification and limits bone resorption, thereby contributing to the prevention of osteoporosis (150, 151).

In addition to peptides, other bioactive compounds found in fermented dairy products -most notably vitamin K₂ (menaquinone)- also play a significant role in supporting calcium bioavailability and promoting bone health, with cheeses fermented using *Propionibacterium* strains being particularly rich in vitamin K₂ (152). During cheese fermentation, vitamin K₂ accumulates as a result of microbial synthesis by specific bacteria, in addition to calcium, contributing to bone health. Vitamin K₂ supports osteoblast proliferation and activity through mechanisms including activation of the Wnt/β-catenin pathway, regulation of oxidative stress, and upregulation of osteoblast- and extracellular matrix-related genes, promoting collagen accumulation and bone mineralization (150, 153–157).

As a result, fermented dairy products emerge as functional foods that contribute to bone health by combining essential nutrients and bioactive compounds with physiological benefits. Their regular consumption appears to support bone mineralization, enhance skeletal strength throughout life. In particular, those containing *Lactobacillus* spp. may offer additional benefits by increasing calcium solubility and activating transport pathways in the intestinal epithelium, thereby helping maintain Bone Mineral Density (BMD) and lowering the risk of osteoporosis.

### 3.6 Neurological effects and mental health

In recent years, research on the interactions between the gut microbiota and the central nervous system has increased. This bidirectional interaction system, called the microbiota-gut-brain axis, plays a critical role in the regulation of a wide range of brain functions, from neurodevelopment to behavioral processes. Fermented dairy products, through the probiotic microorganisms and their metabolites, modulate the composition and activity of the gut microbiota and affect mental health and neurological functions through neuroimmunological and neurochemical mechanisms (158).

Recent studies have increasingly demonstrated that these mechanisms are supported by clinical and experimental findings. In particular, growing evidence from recent research on the gut–microbiota–brain axis suggests potential therapeutic effects of fermented dairy products in neurological and neuropsychiatric disorders, including depression, anxiety, Alzheimer’s disease, and Parkinson’s disease (159, 160). Studies have shown that gamma-aminobutyric acid (GABA)-enriched fermented dairy products improve sleep quality and reduce anxiety-like behaviors by increasing the production of some short-chain fatty acids (SCFA) such as butyrate (161, 162). It has been reported that fermented dairy products provide multiple positive effects such as improving learning and memory functions, alleviating stress responses, modulating the hypothalamic–pituitary–adrenal (HPA) axis and regulating neurotransmitter balance (163, 164).

In an experimental study using Tibetan fermented milk, cognitive impairments were significantly alleviated in transgenic mice with Alzheimer’s disease. After long-term feeding with Tibetan fermented milk, improvements in spatial learning and memory performance were observed; a decrease in amyloid-beta (Aβ) accumulation in the hippocampus and cerebral cortex was detected. It has been reported that fermented dairy products may reduce Aβ accumulation by regulating the gut microbiota (biosis) (80).

Fermented dairy products and metabolites derived from fermented dairy products (e.g., GABA, SCFAs) exert anxiolytic and anti-neuroinflammatory effects through the gut-brain axis. Fermented dairy products help probiotic bacteria to survive without being damaged by stomach acid by providing substances that mitigate the effect of acid in the stomach and facilitate the attachment of probiotics.

Bioactive metabolites derived from fermented dairy products, particularly small bioactive peptides, GABA, SCFAs, exopolysaccharides, and microbial organic acids (e.g., lactic acid and phenyllactic acid), are suggested to regulate key signaling pathways including PI3K/AKT/mTOR and GABA_B receptor-associated cAMP/PKA/CREB signaling, thereby reducing oxidative stress and modulating gut–brain axis activity (165, 166). These effects have also been demonstrated in preclinical mouse models, where fermented dairy-derived bioactive compounds showed neuroactive and metabolic regulatory effects. In a human study on 70 university students, it was observed that anxiety levels decreased with increased consumption of yogurt and cheese. This shows that GABA obtained from fermented dairy products is also effective on humans (167).

Recent studies suggest that dietary components and eating habits may play an important role in the cognitive function of older adults (168). However, evidence regarding the relationship between fermented dairy products and cognitive performance is limited and inconsistent. Although short-term cross-sectional studies generally report a positive relationship between fermented dairy products, particularly cheese consumption, and specific cognitive functions, long-term longitudinal studies do not confirm these findings (169–171). In a large cohort study from the UK Biobank including 54.229 participants reporting cheese consumption once per week, weekly cheese consumption was associated with a reduced risk of dementia; however, this association lost significance after adjustment for dietary and lifestyle confounders (172). This suggests that dietary and lifestyle patterns may be determinant in the observed associations.

**Figure 5:**
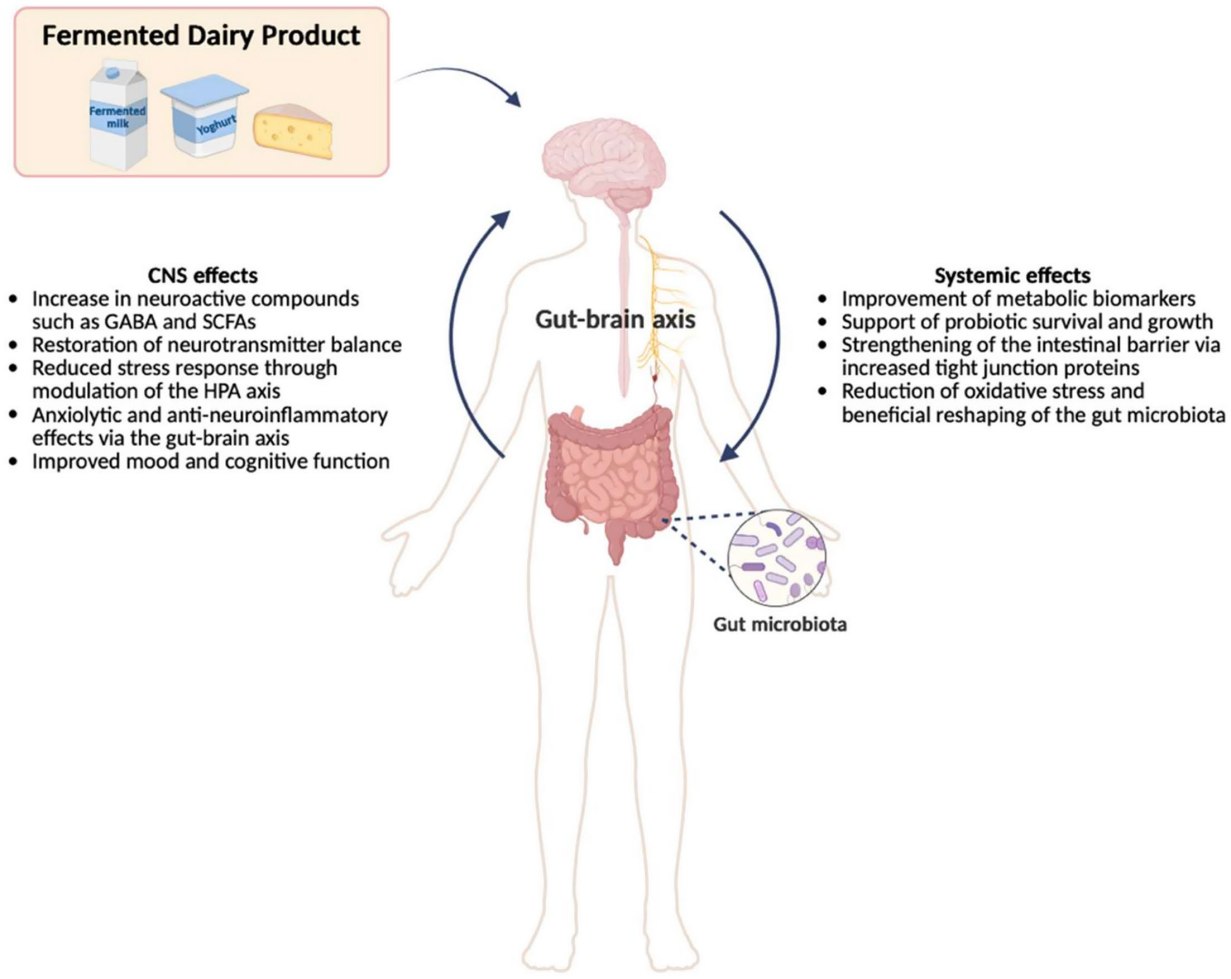
Effects of fermented dairy products on the systemic and central nervous system (CNS) via the gut-brain axis (SCFA: some short-chain fatty acids, GABA: gamma-aminobutyric acid).

In a 4-week randomized controlled crossover trial involving 23 older women (≥70 years) with mild cognitive impairment reported that twice-daily consumption of mold-fermented cheese (Camembert) significantly increased brain-derived neurotrophic factor (BDNF) levels compared with the control period, during which no cheese was consumed (173). Previous epidemiological studies have suggested potential beneficial associations between fermented dairy intake and mental health outcomes, including reduced risk of depressive symptoms and improved psychological well-being. However, findings remain inconsistent across cohorts. In particular, some studies have reported inverse associations between fermented dairy consumption and depression risk, whereas others indicate that these associations are attenuated or lost after excluding cheese intake or adjusting for lifestyle-related confounders (171, 174). A meta-analysis including 8 studies with a total of 83.533 participants reported that fermented dairy food intake was associated with a significantly reduced risk of depression [Odds Ratio (OR) = 0.89, 95% CI: 0.81–0.98]. In subgroup analyses, both cheese (OR = 0.91, 95% CI: 0.84–0.98) and yogurt consumption (OR = 0.84, 95% CI: 0.72–0.99) were significantly associated with lower depression risk; however, no significant effect was observed for excessive intake of fermented dairy products (175) (Figure 5).

In short, the studies mentioned above suggest that fermented dairy products exert neuroprotective, anti-inflammatory, and cognitive function-enhancing effects by regulating the gut microbiota, and that these effects indicate the potential role of fermented dairy products in supporting mental health and preventing neurodegenerative disorders.

## 4 Challenges and limitations

### 4.1 Microbiological and safety concerns

**Figure 6:**
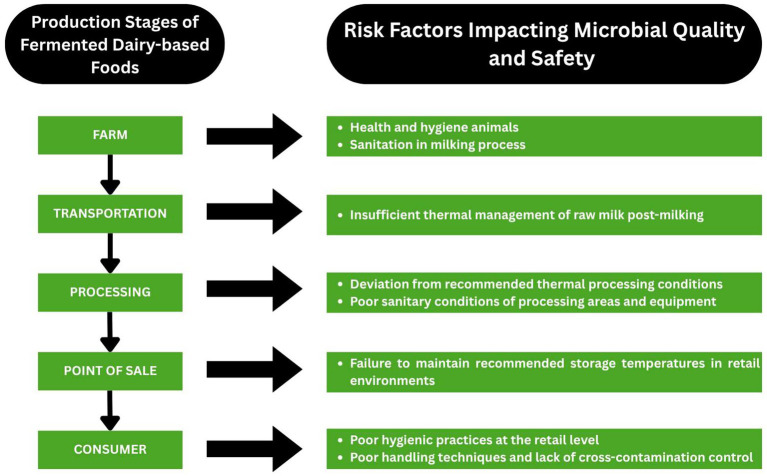
Stages of the dairy supply chain and the relevant microbial and safety risk factors (Created with BioRender.com).

The microbial safety and quality of dairy-based fermented products may affect the combined effects of contamination risks and the likelihood of survival or proliferation of microorganisms and pathogens throughout the entire production and distribution chain, starting from milking and extending to the point of consumption. These risks and microbial dynamics are shaped by factors such as on-farm hygiene and milk handling practices, the conditions under which raw milk is stored and transported to processing facilities, the parameters of the processing itself, and the subsequent handling and storage of finished dairy products at the wholesale, retail, and consumer levels (176). Figure 6 illustrates the key stages of the dairy value chain along with the corresponding risk factors impacting microbiological and safety concerns.

Despite their nutritional benefits, fermented dairy products may pose various health risks, primarily associated with contamination during fermentation processes (91, 177). These risks are largely linked to the formation or presence of harmful compounds, such as biogenic amines and mycotoxins, which are discussed in the following subsections.

#### 4.1.1 Biogenic amines

Biogenic amines (BAs) are nitrogenous compounds produced primarily through microbial decarboxylation of amino acids or through enzymatic reactions involving ketones and aldehydes during fermentation (178). In fermented foods, LAB involved in the fermentation process are primarily responsible for the formation of biogenic amines. These microorganisms synthesize amino acid decarboxylase enzymes, which catalyze the conversion of amino acids into their corresponding amines (179). Although low levels of BAs are typically harmless due to their physiological roles and the activity of intestinal amine oxidases, the accumulation of BAs in food products can pose significant toxicological risks, with symptoms ranging from headaches, flushing, heart palpitations, and dizziness to blood pressure disturbances and, in severe cases, life-threatening effects (180). High concentrations of BAs have been commonly reported in various food items, particularly in fermented products including dairy foods (181). The main biogenic amines generated during the fermentation of these products include putrescine, phenylethylamine, histamine, tyramine, and cadaverine. Among these, histamine and tyramine are of particular concern due to their prevalence and significance in terms of food safety (182).

The presence of BAs in milk and fermented dairy products varies depending on factors such as animal species, heat treatment applied, and product processing, with fermented products generally exhibiting lower levels (183). BA levels in cheeses can vary both among cheese types and across different regions of the same cheese, with levels generally lower in short-ripened cheeses than in longer-ripened cheeses (184). Lactic acid bacteria such as *Enterococcus*, *Lactobacillus* spp., *Leuconostoc*, *Lactococcus*, and *Streptococcus* are important BA producers in cheeses, with *Enterococcus* and lactobacilli species playing a more prominent role, particularly in cheeses produced from unpasteurized milk (185). The presence of histamine at 100 mg/kg, tyramine at concentrations ranging from 100 to 800 mg/kg, and phenylethylamine at 30 mg/kg in foods has been reported to pose potential toxic risks (186). While histamine and tyramine levels above toxic thresholds have been reported in some cheese types (e.g., blue cheese, Parmesan, mish cheese, hard and soft cheeses), BA levels are relatively low in yogurt, ayran, kefir, and some fermented milks. Furthermore, the ability of certain strains of lactic acid bacteria to degrade BAs during storage may contribute to the decrease or stability of these amines over time. Therefore, limiting BA accumulation in cheese production, control of BA-producing microorganisms, appropriate heat treatment, development of hygiene practices, use of non-BA-producing starter cultures, and storage at low temperatures are recommended (181).

#### 4.1.2 Mycotoxins

Mycotoxins are toxic, low-molecular-weight secondary metabolites produced by various fungal species, particularly from the genera *Alternaria*, *Aspergillus*, *Fusarium*, and *Penicillium*. Mycotoxin contamination is a significant concern due to favorable climatic conditions and inadequate agricultural practices, such as poor harvesting, drying, and storage techniques (187). Animal feed, often composed of cereal byproducts, forage, and straw, is particularly susceptible to contamination by multiple mycotoxins. These mycotoxins frequently co-occur in raw feed ingredients, leading to the accumulation of multiple toxins in livestock diets (188). Upon ingestion by ruminants, some mycotoxins are metabolized into derivatives, which are excreted into milk within hours and are classified as a possible human carcinogen. Although certain mycotoxins may be partially detoxified in the rumen, others can bypass this system entirely and remain biologically active (177). Chronic exposure to such contaminants through daily consumption of contaminated dairy products poses significant health risks, including impaired child growth, immune suppression, gut dysfunction, and increased cancer (189).

Given the potential health risks associated with mycotoxin contamination in dairy products, various mitigation strategies have been explored to reduce their presence and biological activity. Among these, fermentation has attracted attention due to its possible role in limiting fungal growth and decreasing mycotoxin bioavailability. However, the extent of detoxification depends on multiple factors, including the microbial strain, fermentation conditions, and the physicochemical properties of the toxins. Certain strains of LAB are known to produce antifungal metabolites, such as lactic acid, phenyllactic acid, hydroxyphenyllactic acid, indole, and bioactive peptides, that can inhibit fungal growth and suppress mycotoxin production. In addition to these antimicrobial compounds, antifungal effects may also result from microbial competition for ecological niches and essential nutrients, as well as from environmental modifications induced by LAB. Furthermore, the ability of bacterial cell wall components, including polysaccharides and peptidoglycans, to bind mycotoxins contributes to detoxification, as it limits their absorption and reduces their harmful effects (190).

Among the bioactive components naturally present in fermented dairy products, peptides have attracted particular attention not only for their nutritional and physiological benefits but also for their potential role in enhancing microbiological safety. Representative examples include lactosericin from lactotransferrin, pediocin-like bacteriocins (Class IIa) from casein, and lactoferricin obtained through the enzymatic hydrolysis of lactotransferrin. These peptides exert antimicrobial activity through selective interactions with microbial cell membranes, disrupting membrane integrity via *barrel-stave*, *carpet*, or *toroidal-pore* mechanisms, ultimately leading to microbial cell lysis and a reduction in contamination risks within food matrices (191). In addition, certain antioxidant peptides present in fermented dairy products, such as Val-Pro-Pro (VPP), Ile-Pro-Pro (IPP), Tyr-Pro-Phe-Pro-Gly (YPFPG), and Leu-Leu-Val-Tyr (LLVY), modulate host cellular stress responses through the Keap1–Nrf2 signaling pathway, thereby improving host resilience and indirectly suppressing undesirable microbial proliferation (192). Although bacteriocins exhibit antimicrobial activity, they are generally susceptible to degradation by gastrointestinal proteases such as trypsin and chymotrypsin. This rapid degradation limits their activity in the gastrointestinal tract, thereby supporting their safe application without significantly disrupting the gut microbiota or inducing systemic immune responses (193). Through these combined molecular mechanisms, bioactive peptides present promising opportunities for integration into microbiological safety strategies, complementing their nutritional value with a protective role against microbial contamination.

**Figure 7:**
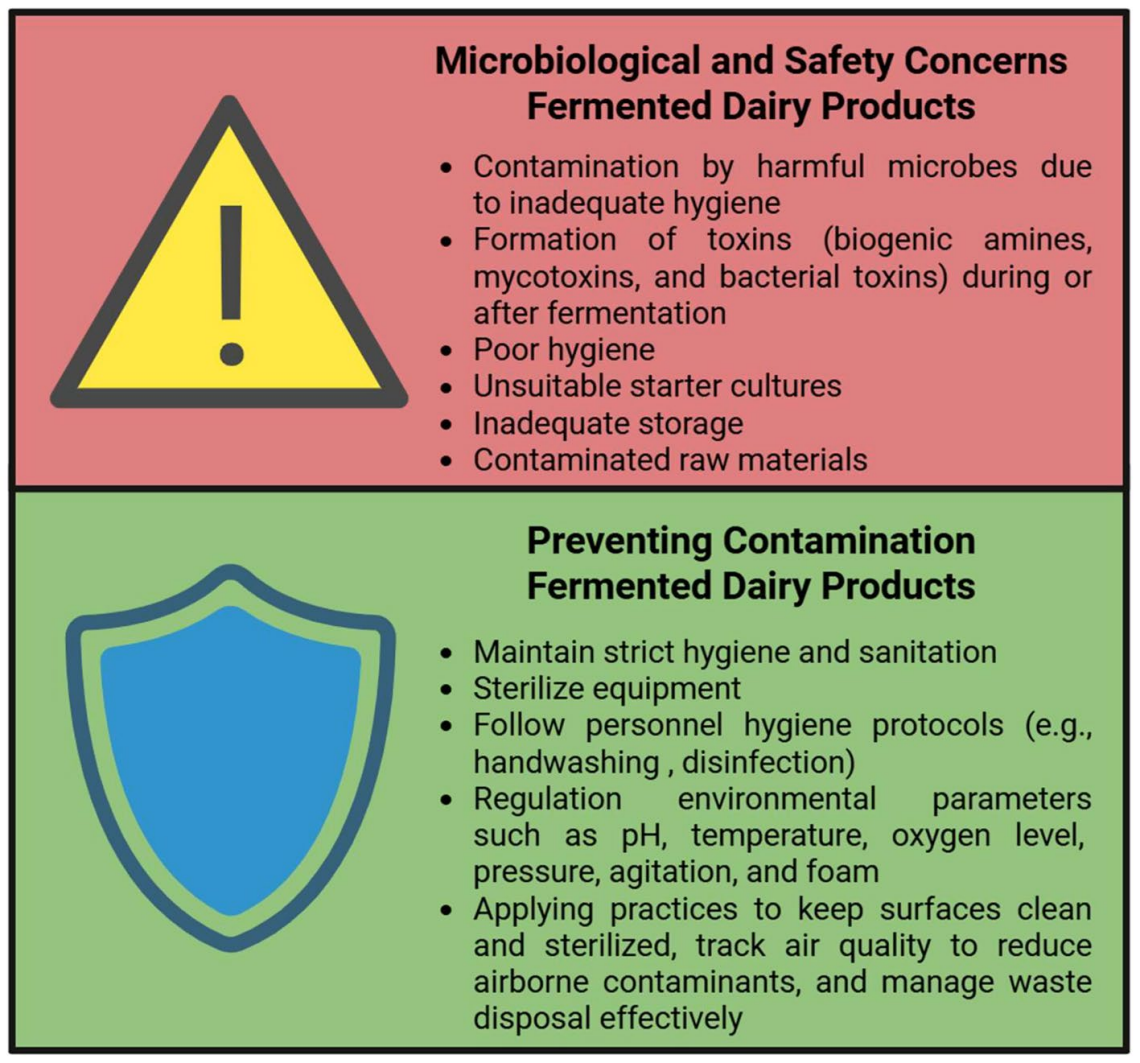
Summary of risks, causes, and preventive measures in fermented dairy production (Created with BioRender.com).

Microbiological and safety concerns in fermented dairy products can arise from microbial toxins such as mycotoxins, which can be formed as a result of inadequate hygiene practices and microbial contamination during the production process. The emergence of these toxins is also associated with factors such as inappropriate starter culture selection, inadequate storage conditions, and the use of contaminated raw materials. Therefore, maintaining strict hygiene and sanitation practices throughout production is crucial. Safe production requires the control of raw materials, sterilization of equipment, compliance with hygiene rules by personnel, regulation of environmental parameters such as pH, temperature, and oxygen in the production facility, and monitoring of air quality. Figure 7 and Table 3 summarize the microbiological and safety concerns about fermented dairy products.

**Table 3:** Challenges and limitations about microbiological and safety concerns of fermented dairy products.

Challenge/Limitation	Description	Impact on safety and quality	Possible solutions/remarks	References
Contamination Risk during Production	Difficulty in maintaining strict hygiene throughout fermentation and processing	Risk of contamination by pathogens and spoilage microorganisms	Implementation of GMP and HACCP systems	(227)
Variability of Starter Cultures	Genetic and phenotypic differences in starter cultures leading to inconsistent fermentation	Variable product quality and potential safety issues	Use of well-characterized, standardized starter cultures	(228)
Detection of Pathogens and Toxins	Limited sensitivity and specificity of routine microbiological tests	Undetected pathogens/toxins may pose health risks	Adoption of advanced detection methods (PCR, biosensors)	(177)
Formation of Biogenic Amines	Difficult to control due to diverse microbial populations and fermentation conditions	Toxic effects if accumulated in high amounts	Selection of starter cultures with low decarboxylase activity	(180)
Antimicrobial Resistance Spread	Potential transfer of resistance genes from starter or contaminant microbes	Reduced effectiveness of antibiotics in treatment of infections	Monitoring resistance and limiting antibiotic use	(229)
Storage and Shelf-Life Limitations	Changes in microbial populations during storage affecting product safety and quality	Spoilage, toxin production, and pathogen growth	Proper cold chain management and packaging innovations	(230)
Regulatory and Standardization Issues	Lack of unified global standards for fermented dairy microbiological safety	Variable safety levels depending on region and producer	Development and harmonization of international standards	(231)
Consumer Awareness and Education	Limited knowledge about safety risks and proper handling of fermented dairy products	Increased risk of foodborne illness due to improper storage or consumption	Public education campaigns and clear labeling	(232)

GMP, Good Manufacturing Practices; HACCP, Hazard Analysis and Critical Control Points.

#### 4.1.3 Probiotics

Although probiotics in fermented dairy products such as cheese are widely studied, labeling and quality control issues remain a significant concern, particularly in probiotic-rich dairy products. In recent years, the global probiotic market has grown in parallel with the increasing interest in the health benefits of probiotics; however, challenges persist regarding the accuracy and reliability of labeling (194). For example, a study investigating labeling issues in the probiotic market in Ghana reported that such problems were more prevalent in probiotic foods than in probiotic supplements (194). According to this study, the compliance rate was 74% for probiotic supplements, while it was 44% for probiotic foods. Common problems included the lack of essential information such as viable cell counts (CFU), failure to specify the probiotic strain present in the product, lack of scientific evidence supporting health claims, and the inclusion of incorrect or non-existent bacterial names on labels. Furthermore, while some probiotic foods specified the genus, others did not indicate the species or strain level (194).

In addition, discrepancies between declared and actual microbial content have been reported in commercial probiotic products. For instance, a study conducted in China found that the total viable cell counts measured in probiotic products did not match the values declared on the label (195). Similarly, a study in Canada evaluating 22 commercial probiotic products reported that although expected bacterial counts were provided in 60% of the products, labeling inaccuracies were common; in 32% of the products listing specific microorganisms, at least one organism name was misspelled. Moreover, only 27% of the products making specific claims about viable microorganisms met the stated label claims, and very few products accurately reflected their contents (196). These findings emphasize the need for stricter quality control, accurate labeling, and reliable verification of probiotic content, particularly in probiotic-rich dairy products.

### 4.2 Technological and production challenges

#### 4.2.1 Strain selection and stability

The effectiveness and consistency of dairy-based fermented foods is largely dependent on the selection of microbial strains, particularly LAB, bifidobacteria, and yeasts (197–199). While a significant number of strains have been designated with Qualified Presumption of Safety (QPS) status, it is important to highlight that their functional properties tend to vary considerably across species and even within strains (198). The critical challenges that should be addressed include the maintenance of genetic and metabolic stability during large-scale fermentation, ensuring resistance to bacteriophage attacks, and preserving viability during storage and distribution (198). Furthermore, strain interactions in mixed cultures, such as those found in kefir or traditional starters, have the potential to complicate fermentation dynamics, thereby resulting in variability in product quality and health-related properties (197, 199). It is also critical that probiotic strains are able to survive gastrointestinal transit and colonize the gut in order to provide health benefits (197). Standardisation of starter cultures, which have been found to be both stable and reproducible, in addition to the utilisation of genomic and metabolomic tools for strain characterisation, remains a pressing technological necessity (198).

#### 4.2.2 Scalability vs. artisanal methods

The main challenge in the production of fermented dairy foods is balancing industrial scalability with the preservation of artisanal characteristics. It is evident that traditional fermentation practices, including back-slopping and the utilisation of complex natural starter cultures, play a pivotal role in promoting rich microbial diversity. However, reproducing these naturally complex microbial diversity at an industrial scale is difficult due to their inherent variability, sensitivity to environmental fluctuations, and susceptibility to contamination (21, 200).

In contrast, large-scale industrial fermentation prioritizes safety, efficiency, and product consistency, often relying on standardized starter cultures. While this approach enhances predictability and quality control, it can inadvertently reduce microbial diversity and lead to a loss of traditional flavors and functional metabolites such as exopolysaccharides, peptides, and short-chain fatty acids. The simplification of microbial diversity has been associated with decreased probiotic potential and narrower metabolic functionality compared to traditional mixed cultures (200).

In order to bridge the gap between artisanal authenticity and industrial efficiency, innovative strategies are required. Emerging solutions include controlled co-culture fermentations, which allow for predictable microbial interactions and enhanced metabolite diversity; precision fermentation, which utilizes genomic and metabolomic tools to guide strain performance and stability; and hybrid production models that integrate the microbial richness of traditional starters into controlled industrial environments (201, 202). The integration of these approaches, supported by real-time monitoring, adaptive process control, and multi-omics data, has the potential to facilitate scalable production of fermented dairy products without compromising their sensory richness and functional health benefits (201). However, the capacity of hybrid and precision-based production models to completely maintain artisanal functional metabolite profiles in large-scale industrial settings remains limited. It is important to note that variability in microbial interactions, substrate availability, and environmental parameters may still result in variations in bioactive compound production across batches. Furthermore, the implementation of such systems necessitates considerable technical expertise, substantial infrastructure investment, and regulatory validation, which may result in a limited adoption rate. Consequently, while hybrid approaches may offer a promising transitional strategy, their long-term effectiveness in replicating traditional fermentation complexity on a large scale remains context-dependent.

### 4.3 Nutritional and consumer-based limitations

#### 4.3.1 Lactose intolerance and dairy allergies

The consumption of fermented dairy products may be limited among individuals affected by lactose intolerance and milk protein allergies, despite the well-documented health-promoting potential of these foods (203). Although fermentation partially reduces residual lactose content, concentrations may remain sufficient to provoke symptoms in highly sensitive individuals (204).

Cow’s milk protein allergy (CMPA) is an immune-mediated hypersensitivity reaction primarily directed against casein and *β*-lactoglobulin. Although fermentation may partially hydrolyse these proteins and generate smaller peptides, allergenic epitopes may persist and residual immunoreactivity may remain. Consequently, traditional fermented dairy products may not be suitable for individuals with CMPA, particularly infants and children (205).

Recent research highlights the development of hypoallergenic and lactose-free fermented alternatives, including enzymatically hydrolysed dairy beverages and microfiltered whey-based formulations (206) (Molina et al., 2023). These approaches aim to preserve the nutritional value and functional bioactivity associated with fermented products while improving digestibility and consumer tolerability among individuals with lactose intolerance or milk protein sensitivities.

#### 4.3.2 Sodium content in fermented dairy products

Cheese and other salt-cured dairy products are valuable sources of high-quality protein, calcium, and bioactive peptides, contributing significantly to human nutrition (Faccia and Natrella, 2024). However, their relatively high sodium content has raised concerns regarding the risk of hypertension and cardiovascular disease, which remain major global public health issues (Hoppu et al., 2017). Salt is commonly added during cheesemaking to control microbial growth, regulate moisture, and contribute to flavour and texture development. Nevertheless, excessive sodium intake has been associated with adverse health outcomes, particularly in populations with high cheese consumption (Leclercq-Perlat et al., 2024).

The dairy sector therefore faces the challenge of maintaining desirable product quality while aligning with evolving nutritional guidelines that encourage sodium reduction (Hoppu et al., 2017). Several sodium-reduction strategies have been explored, including partial substitution of sodium with potassium or calcium salts, the use of flavour-enhancing starter cultures, and technological modifications in cheese ripening processes (Leclercq-Perlat et al., 2024). However, implementation of these strategies remains limited by consumer acceptance, regulatory constraints, and the potential development of off-flavours or altered textures in reduced-sodium cheeses (207). Recent studies suggest that multi-salt systems and adaptive microbial cultures may help retain desirable sensory profiles while reducing sodium levels by up to 30–40% without compromising product safety or ripening dynamics (Govindasamy-Lucey et al., 2023; Leclercq-Perlat et al., 2024). Consequently, effective sodium reduction in dairy production requires integrated technological and consumer-centred strategies to meet modern nutritional goals while maintaining traditional product characteristics (Faccia and Natrella, 2024).

## 5 Conclusion

Dairy-based fermented foods are a wide range of traditional and modern products that have significant nutritional and functional properties. As well as their role in preserving and improving the sensory properties of food, these foods may contribute to human health by modulating the gut microbiota, supporting immune function, improving lactose digestion, regulating blood pressure and lipid metabolism, providing antioxidant activity and enhancing mineral bioavailability. These effects are largely associated with the metabolic activity of lactic acid bacteria and related microorganisms, which produce bioactive compounds such as peptides, exopolysaccharides and short-chain fatty acids. Several of these metabolites have been linked to biological mechanisms, including the inhibition of angiotensin-converting enzyme, the modulation of inflammatory signalling pathways, and the improvement of nutrient absorption.

Despite growing evidence from experimental and preclinical studies, translating these findings into consistent health outcomes and validated functional claims remains challenging. Variability in host-related responses, alongside differences in microbial strains, fermentation conditions, and product composition, complicates the standardisation and generalisation of reported benefits. Additionally, the dairy industry faces technological and regulatory challenges concerning product standardisation, quality control, and the rigorous scientific substantiation required to validate probiotic efficacy and functional food claims.

Future research should focus on improving the characterisation of fermented dairy products, particularly through more precise identification of microbial strains and associated bioactive compounds. In addition, further studies are needed to better understand the mechanisms underlying their potential health effects, especially in relation to gut microbiota modulation and metabolic outcomes. Well-designed human studies are required to clarify the relationships between fermented dairy consumption and health-related outcomes, taking into account variability in product composition and host-related factors. Addressing these gaps will strengthen the evidence base for fermented dairy products as functional foods and support the development of evidence-based nutritional and public health strategies.
